# Influence of sample positioning and solution volume on distinguishing sonolysis from piezocatalysis in ultrasonic dye degradation

**DOI:** 10.1016/j.ultsonch.2026.108049

**Published:** 2026-09-10

**Authors:** Mohit Singhal, Akshay Gaur, Brajesh Kumar Nagar, Dhiraj Kumar Singh, Satyanarayan Patel

**Affiliations:** Department of Mechanical Engineering, Indian Institute of Technology Indore, 453552, India

**Keywords:** Piezocatalysis, Sonolysis, Acoustic field, NBT

## Abstract

Piezocatalysis has emerged as an optimistic mechanically assisted catalytic process for the degradation of organic pollutants, in which ultrasonic waves stimulate the piezoelectric material and activate its catalytic activity. Laboratory-scale piezocatalytic investigations commonly utilize an ultrasonicator, which produces a spatially varying acoustic field, while sonolysis, contributes to variations in degradation efficiency. The radicals produced through sonolysis also contribute to pollutant degradation, thereby introducing uncertainty in the assessment of the piezocatalyst’s apparent piezocatalytic activity. The present study investigates the contribution of sonolysis in the piezocatalysis process by examining degradation at various locations within the ultrasonicator. The outcomes of the study demonstrate that there is inconsistency in degradation with spatial positioning. Furthermore, a critical minimum pollutant volume was identified above which consistent results were obtained for sonolytic dye degradation. The findings from the investigations strongly recommend mandatory steps of optimizing sample positioning and pollutant volume prior to any piezocatalytic investigation that need to be followed to ensure repeatability and isolate sonolysis contributions.

## 1 Introduction

Sonochemistry is a well-established scientific field that leverages ultrasound principles and has extensive applications in industrial, environmental, and biomedical areas [1], [2]. Sonochemistry uses cavitation to create localized hotspots characterized by high-temperature and pressure zones, consequently facilitating its adoption in extraction, crystallization, synthesis, materials fabrication, and non-surgical therapies [3]. In recent years, the degradation of pollutants using mechanical energy has attracted increasing attention [4], [5], [6], [7]. Among the many mechanically assisted catalytic processes, piezocatalysis has emerged as a promising catalytic process, with the potential to utilize sustainable energy sources such as wind, noise, and fluid motion [8]. Being a progressive research field, notable efforts are being made to enhance the efficiency of piezocatalysts. Some of the strategies include controlling the morphology and particle size, varying the doping concentration, introducing oxygen vacancies, and constructing heterojunctions [9], [10], [11], [12]. These strategies intend to promote the charge separation and promote the formation of reactive oxygen species (ROS), thereby accelerating degradation kinetics. Overall, the piezocatalytic process is sustainable, reusable, highly efficient, and economically advantageous compared with conventional catalytic processes [13]. Although piezocatalysis can utilize low-frequency mechanical stimulation for its operation, its practical translation to sustainable mechanical energy sources remains challenging, as most laboratory-scale investigations rely on ultrasonication to provide the mechanical stimulation required to activate the piezoelectric material. Most experimental studies on piezocatalysis continue to employ conventional ultrasonic baths operating at frequencies of ∼ 30–50 kHz [13], [14]. Ultrasonic waves can induce acoustic cavitation, which independently contributes to dye degradation through sonolysis process.

Ultrasonicator in working provides a spatially distributed acoustic field within the water bath and raises a fundamental question: *i.e*, when a piezocatalyst is used for pollutant degradation under the ultrasonic waves generated by an ultrasonicator, is the observed degradation exclusively due to piezocatalytically generated surface charges, or do cavitation-induced effects (sonolysis) also contribute throughout the sonicated region? If so, how can the sonolytic contribution be minimized to determine the apparent piezocatalytic activity of the piezocatalyst? Moreover, the extent of self-degradation caused by increased temperature can be minimized by maintaining a constant low bulk temperature using a circulating cooling system [15].

To observe the influence of sonolysis in piezocatalytic dye degradation and minimize its effect, the present study employs ferroelectric NBT powder to investigate piezocatalytic dye degradation. Clarifying the distinction between acoustic cavitation-induced and piezocatalytic degradation is essential for accurately interpreting experimental results, optimizing reactor design, and assessing the most potential of mechanically driven catalysis for large-scale environmental remediation.

To the best of the authors' knowledge, no previous study has systematically proposed a strategy to minimize sonolysis and asses the apparent piezocatalytic contribution during piezocatalysis. Accordingly, the manuscript first introduces the fundamental principles of mechanically assisted dye degradation, clearly distinguishing sonolysis, sonocatalysis, and piezocatalysis. Building on this foundation, experiments of sonolysis and piezocatalysis with NBT powder were performed.

## 2 Mechanical-assisted processes

### 2.1 Sonolysis process

**Fig. 1:**
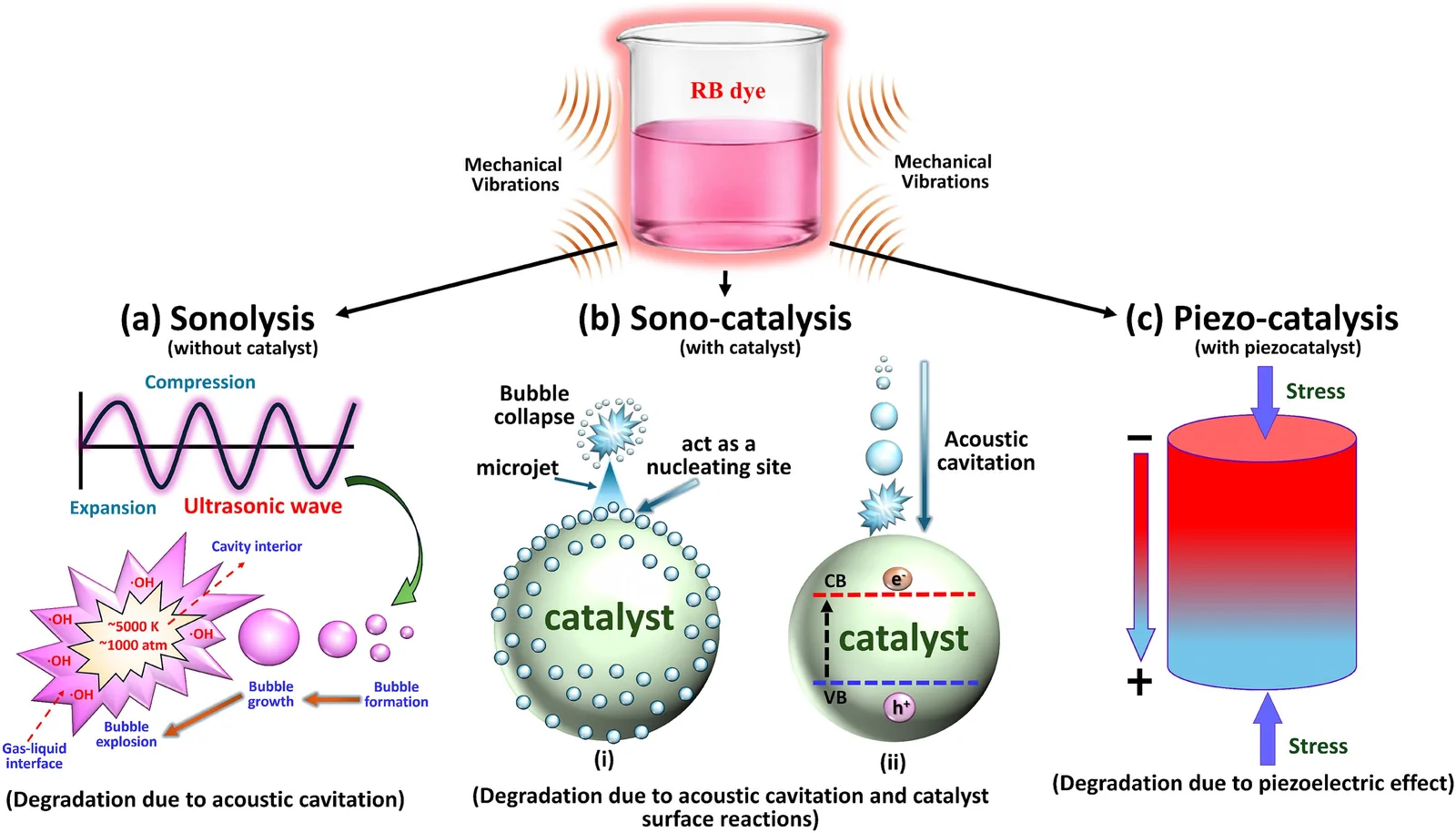
Schematic illustration of ultrasound-assisted dye degradation mechanisms: (a) Sonolysis: acoustic cavitation producing localized high temperature and pressure, leading to radical formation and pollutant breakdown, (b) Sonocatalysis: (i) catalyst promoting nucleating sites, (ii) acoustic cavitation helping electron excitation in catalyst and generation of electron-hole pairs, and (c) Piezocatalysis: stress-induced piezoelectric polarization and charges on catalyst's surface .

Sonochemistry underlies dye degradation under ultrasonic irradiation via acoustic cavitation [16]. As illustrated in Fig. 1(a), alternating compression and rarefaction cycles generated by the ultrasonic transducer cause dissolved gases and pre-existing nuclei in the aqueous medium to grow into cavitation bubbles via rectified diffusion [17]. The moment the bubbles attain a critical size, there is rapid inertial collapse leading to the formation of localized transient hotspots having a temperature of around 5000 K and pressure of 0.1 GPa [18]. It is under such conditions that water molecules fragment to give radicals that include hydroxyl (•OH) and hydrogen (•H), and other oxidants like hydrogen peroxide (H_2_O_2_) and hydroperoxyl radicals (•HO_2_) [19]. These reactive radicals then diffuse into the bulk fluid, where they react with the dye molecules [20].

### 2.2 Sonocatalysis process

Sonocatalysis is a specialized branch of sonochemistry wherein ultrasonic waves act with a solid catalyst to increase chemical reactions [3]. The difference between sonolysis and sonocatalysis is that in sonolysis, pollutant degradation occurs solely due to acoustic cavitation, whereas, in sonocatalysis acoustic cavitation is combined with the catalytic activity of a solid material i.e, sonocatalyst to enhance degradation efficiency [3]. During ultrasonic irradiation, cavitation bubbles generated preferentially nucleate on the catalyst surface owing to its roughness, pores, defects, and trapped gas pockets [21], [22], [23]. Their asymmetric collapse produces localized microjets, shock waves, and transient high-temperature and high-pressure regions [3], [24]. The primary factors affecting the cavitation process include dissolved gases in the liquid, local temperature and pressure induced by acoustic waves, the liquid's physical properties, and acoustic power/frequency [25], [26], [27], [28], [29]. Thus, the catalyst primarily serves as a cavitation promoter and reaction facilitator, while acoustic cavitation remains the dominant driving force for degradation. The semiconducting properties of the catalyst may also contribute to pollutant degradation, in which cavitation excites electrons and generates electron-hole pairs that participate in surface redox reactions, as shown in Fig. 1(b). However, this contribution is secondary, and acoustic cavitation remains the primary driving force [3].

### 2.3 Piezocatalysis process

The piezocatalysis process works on the principle of piezoelectric effect for which the catalyst must be non-centrosymmetric in nature [30]. For continuous functioning, continuous periodic mechanical stimulation is required such that catalyst undergo electric polarization and generates charged surface. The charged surfaces subsequently participate in redox reactions, producing reactive species that degrade pollutant molecules into simpler compounds. Based on the state-of-the-art literature, two plausible mechanisms have been proposed to explain the piezocatalytic degradation process [31] (i) the screening charge theory and (ii) the energy band theory.

**Fig. 2:**
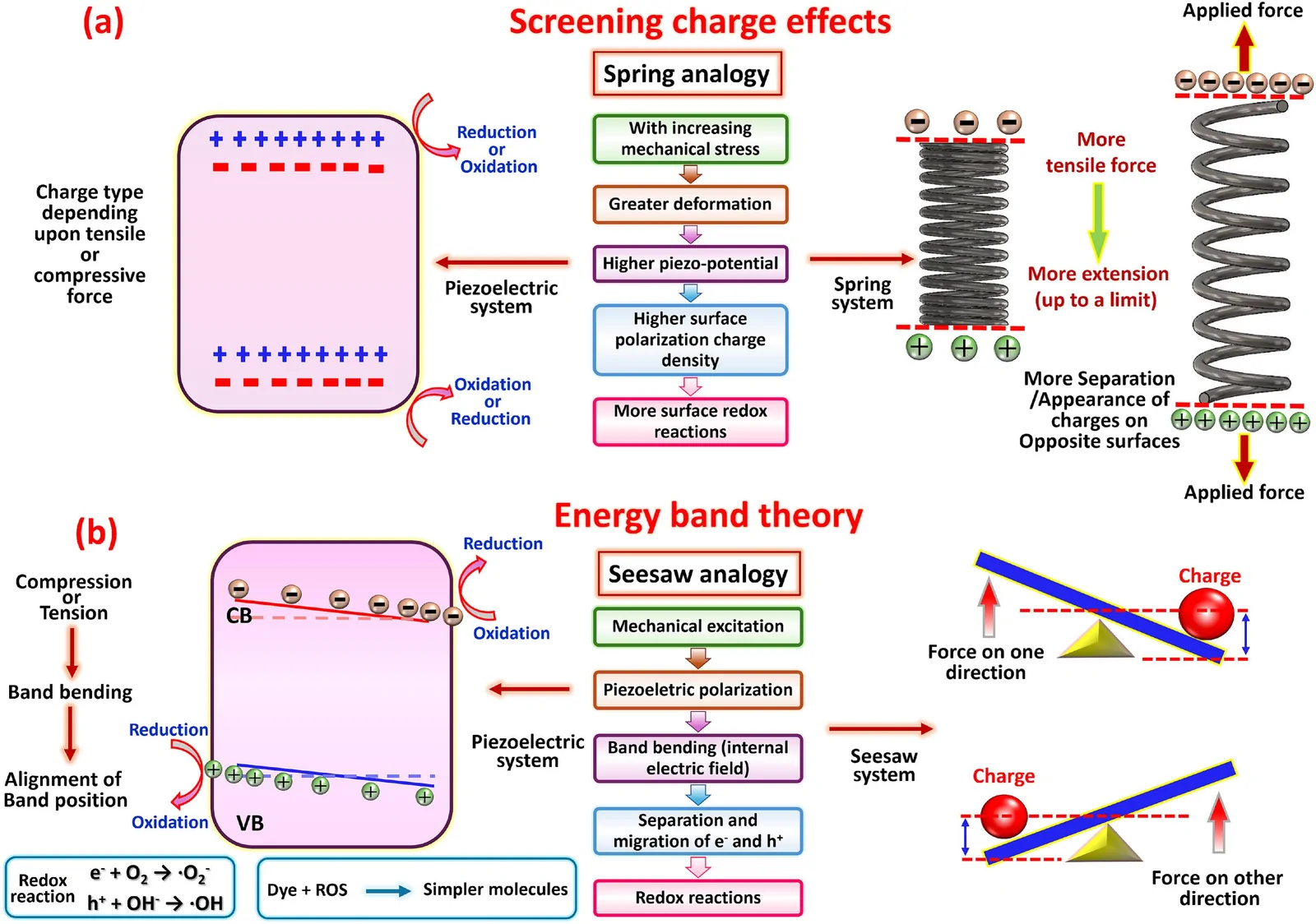
Schematic representation of piezocatalysis mechanism under mechanical stimulation: (a) screening charge redistribution caused by tensile/compressive forces leading to surface redox reactions, illustrated using spring system analogy, and (b) energy band bending and alignment facilitating charge separation, illustrated using a seesaw analogy.

*Screening charge theory:* The screening charge mechanism focuses more on the role of piezo-potential generated by mechanical vibrations and its associated surface screening behaviour. Here, the piezo-potential refers to the electric potential generated when mechanical deformation displaces the positive and negative charge centers, producing polarization and charge separation between the opposite surfaces of the material. Furthermore, the magnitude of the generated piezo-potential must be sufficient to satisfy the redox potential required for the reaction, thereby determining the capability of the piezocatalyst to drive redox reactions. To maintain electrostatic equilibrium during periodic mechanical vibrations, the charges are compensated by oppositely charged ions from the surrounding medium, giving rise to the screening charge effect [32], [33]. As illustrated in Fig. 2(a), the screening charge mechanism can be explained using the analogy of a spring. When a greater tensile or compressive force is applied, the spring undergoes larger elastic deformation. Similarly, increasing mechanical stress leads to greater deformation of the piezoelectric material, generating a larger piezo-potential and a higher surface polarization charge density. For keeping the system in electrostatic equilibrium, more charges of opposite nature would be adsorbed on the surface of the system as screening charges. When the stress is released, there is reduction in piezo-potential, redistribution of screening charges, and continuation of redox reactions cycle by cycle.

*Energy band theory:* According to the energy band theory, mechanical excitation alters the electronic band structure of the piezoelectric material [34]. The resulting band bending facilitates the separation and transport of charge carriers, making sure that the conduction band (CB) and valence band (VB) become aligned with the redox potentials necessary for decomposition of pollutants. As illustrated in Fig. 2(b), this mechanism can be described using the analogy of a seesaw. Under mechanical stimulation, the redistribution of negative and positive charges is analogous to the tilting motion of the seesaw, where one side is elevated while the other is lowered. Such charge redistribution allows reduction reactions at CB and oxidation reactions at VB, thereby enabling efficient piezocatalytic degradation. However, the source of active charge carriers, mechanical stimulation or intrinsic lattice defects is still being questioned in some cases [35].

Although sonolysis, sonocatalysis, and piezocatalysis may all use ultrasonic irradiation as the mechanical energy source, their fundamental degradation mechanisms differ. In sonolysis, pollutant degradation is driven solely by acoustic cavitation, where the collapse of cavitation bubbles generates ROS. Sonocatalysis employs a solid catalyst that can enhance cavitation-induced reactions by providing heterogeneous nucleation sites and promoting reactions at the solid-liquid interface [36]. Meanwhile, piezocatalysis requires a non-centrosymmetric piezoelectric material, in which mechanical deformation generates surface polarization charges that participate in redox reactions. Furthermore, studies by researchers provide another essential element for piezocatalytic dye degradation applications, showing that even in piezoelectric ceramics undergoing mechanical vibrations, sonocatalysis contributes to some extent [37], [38]. The contribution of these processes is influenced by both the frequency and power of the ultrasound [39], [40], [41].

A study shows that at low-frequency ultrasound (<100 kHz), the mechanical effect is the dominant factor, whereas at high frequencies, sonochemical radicals are generated [42]. Consequently, distinguishing the individual contributions of sonolysis, sonocatalysis, and piezocatalysis experimentally remains challenging, particularly under identical ultrasonic operating conditions.

Therefore, the present work focuses on identifying optimized reactor position and solution volume that minimize sonolysis interference by systematically studying degradation, rather than attempting to quantify the individual contributions of sonocatalysis and piezocatalysis. The term piezocatalysis will be used in the investigations in the preceding sections because NBT is a well-known piezoelectric material. As demonstrated by previous researchers, the contribution of sonocatalysis to piezocatalytic degradation is low and is neglected.

## 3 Experimental section

### 3.1 Material (catalyst) synthesis and characterization

Na_0.5_Bi_0.5_TiO_3_ (NBT) ceramic powder was synthesized by solid-state reaction. The precursors Na_2_CO_3_ (99.5 %), Bi_2_O_3_ (99.975 %), and TiO_2_ (99.6 %) (all from Alfa Aesar, Massachusetts, USA) were weighed in stoichiometric ratios and ball-milled for 12 h in ethanol. The milled powder was then dried and subjected to a calcination temperature of 850 °C (2 h), maintaining a ramping rate of 5 °C/min. The structural properties of the synthesized NBT ceramics were studied using X-ray diffraction (XRD) on an Empyrean X-ray diffractometer (Malvern PANalytical). The vibrational modes were observed using Raman spectroscopy (Horiba LabRAM HR Evolution) with 633 nm laser excitation. The microstructure of NBT powder was seen by employing field emission scanning electron microscopy (FESEM) (FE-SEM JEOL JSM-7610 plus). Dielectric properties were evaluated over the temperature range 30–350 °C and frequency range 100  Hz-1  MHz using an LCR meter (NF Corporation ZM2376). Pyroelectric current was measured with a Keithley 6517B and a custom-built step-up, at a heating rate of 5 K/min. The piezoelectric coefficient (*d_33_*) of the poled samples was measured using a Quasi-Static Piezo d33 Meter (PKD3-2000-F10N) under an alternating force of 0.25 N at a frequency of 110 Hz.

### 3.2 Dye degradation investigation

Rhodamine dye (RB) was used for investigations, in which mechanical vibrations were generated using an ultrasonicator (Bandelin Sonerex : RH 255H, 35 kHz, 160 W, 5.5 L). The water in the ultrasonicator was continuously circulated during both the sonolysis and piezocatalysis experiments to maintain its temperature at room temperature. Dye degradation was quantified by subtracting the absorbance at the RB dye's signature wavelength from the absorbance at 15-minute intervals. Absorbance was measured using a UV-Vis spectrophotometer (UV-Vis double-beam spectrophotometer YS-674).

## 4 Results and discussions

### 4.1 Degradation of RB dye-filled glass reactor

**Fig. 3:**
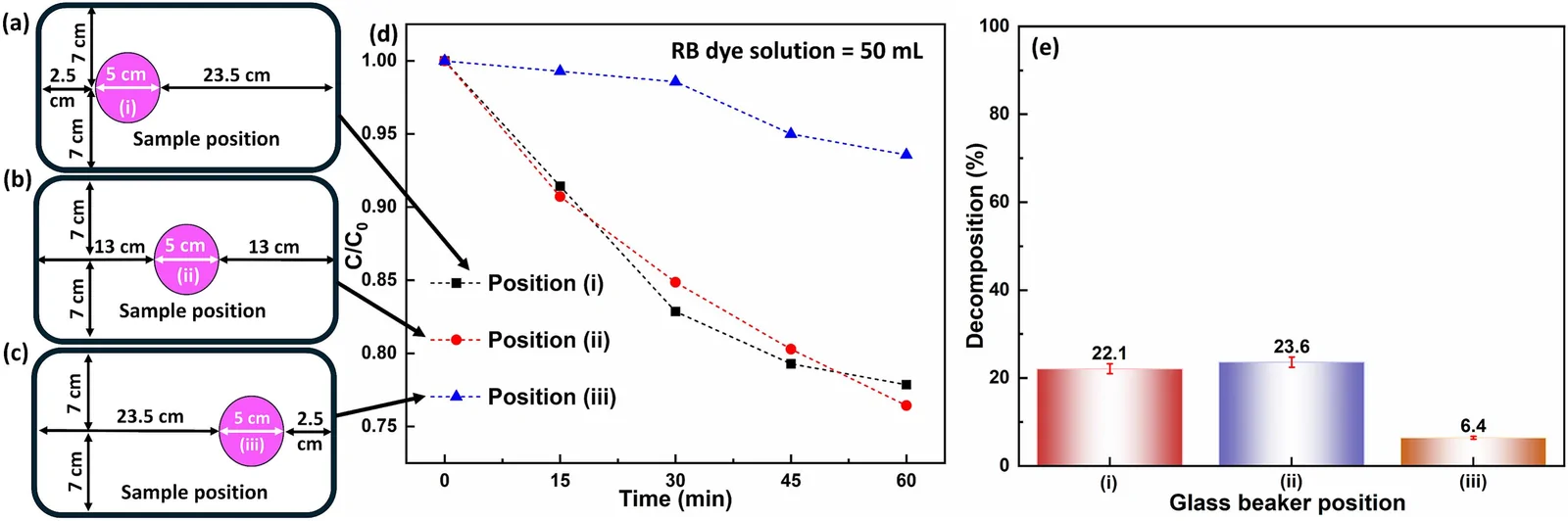
(a-c) Schematic illustration of the ultrasonicator and sample positions, (d) decomposition ratio, and (e) % decomposition of RB dye solution at positions (i), (ii), and (iii) in 60 min.

**Fig. 4:**
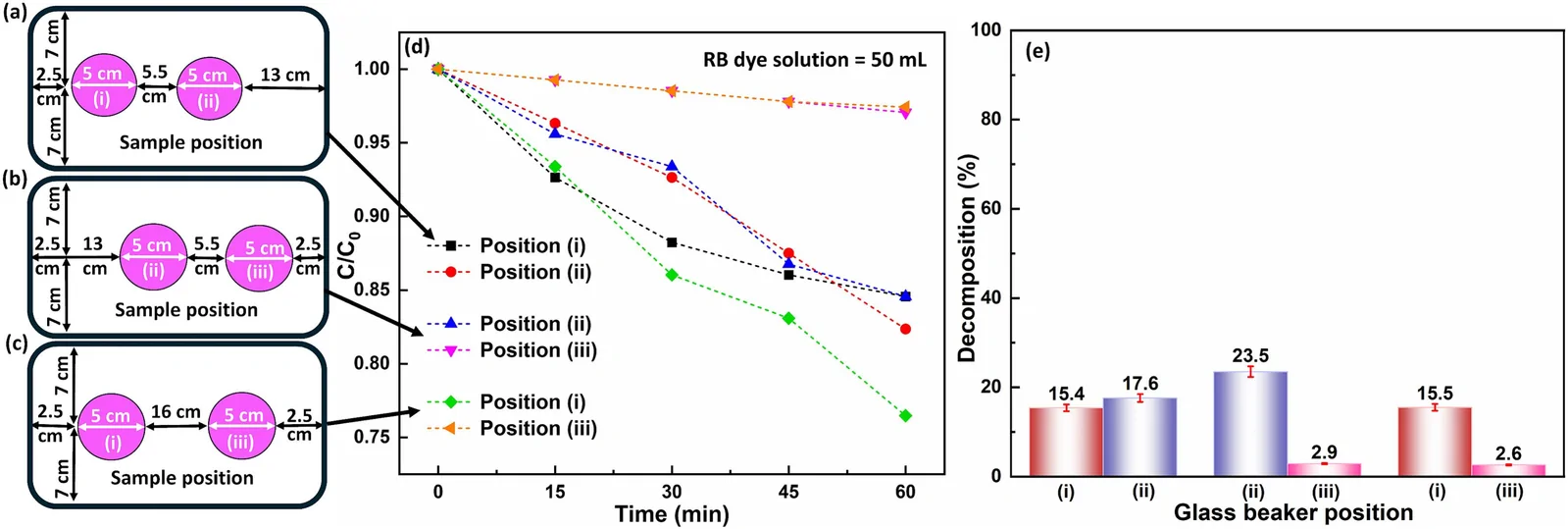
(a-c) Schematic illustration of the ultrasonicator and sample positions, (d) decomposition ratio, and (e) % decomposition of RB dye solution at positions (i)/(ii), (ii)/(iii), and (i)/(iii) in 60 min.

The degradation of RB dye was first investigated in the absence of NBT powder using a 100 mL glass beaker containing 50 mL dye solution. To examine the influence of the acoustic field, the ultrasonic bath was divided into three positions, denoted as (i), (ii), and (iii) (Fig. 3(a)-(c)). The degradation profiles are shown in Fig. 3(d), while the final degradation after 60 min is summarized in Fig. 3(e). The results demonstrate a strong dependence of degradation on the beaker's position relative to the reactor position. Positions (i) and (ii) revealed comparable degradation efficiencies, whereas position (iii) showed the lowest degradation, validating that the acoustic field inside the ultrasonic bath is spatially non-uniform. The investigation was further carried out by placing the two beakers simultaneously at positions (i)/(ii), (ii)/(iii), and (i)/(iii) as presented in Fig. 4(a)-(c). Although the % degradation changed depending on the beaker combination, the same trend was observed, with position (iii) again showing the lowest degradation. This observation can be attributed to the presence of two beakers, which redistribute the acoustic energy within the ultrasonicator bath.

**Fig. 5:**
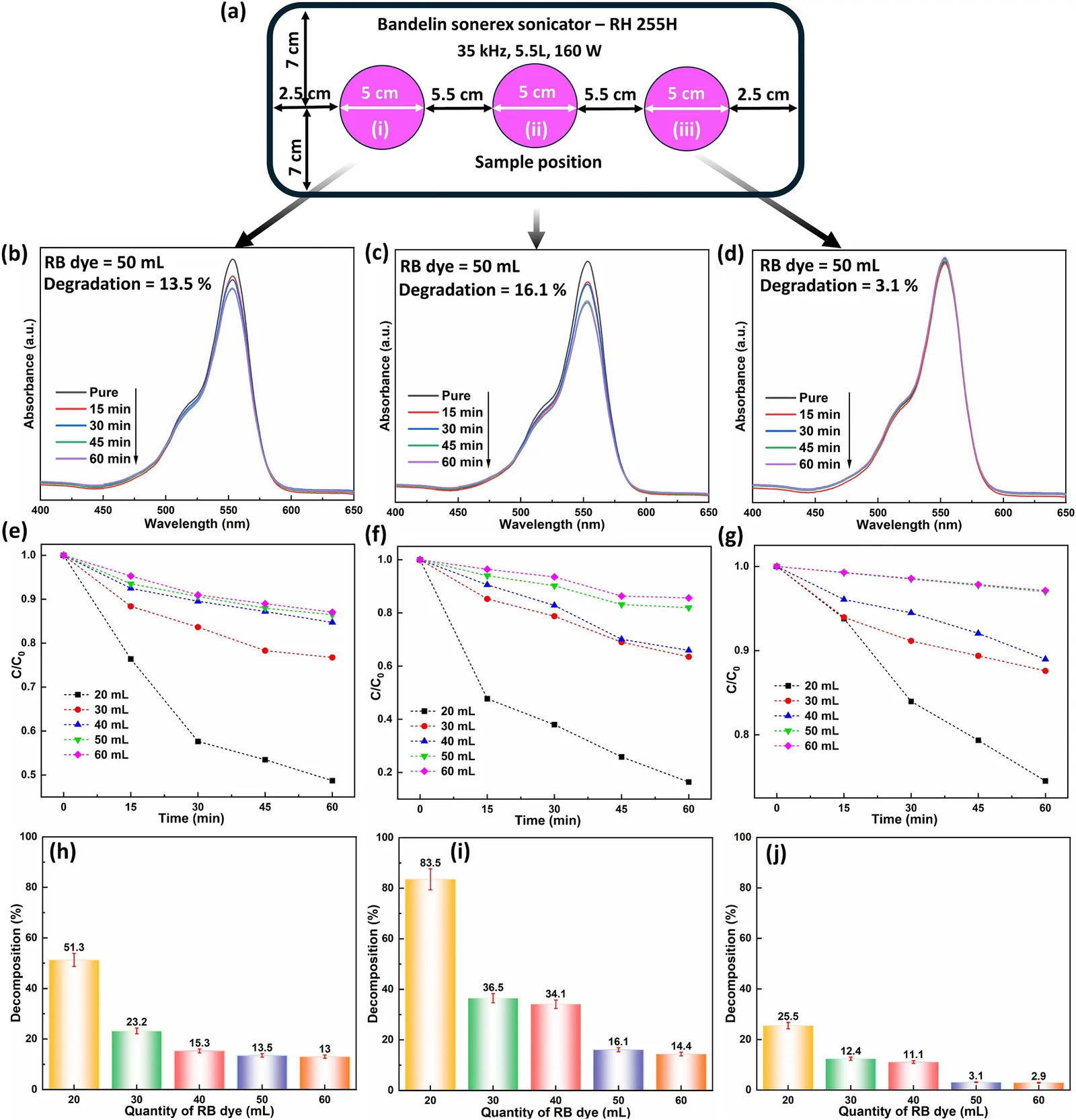
(a) Schematic representation of beaker simultaneously placed at positions (i), (ii) and (iii), Absorbance spectrum of RB dye when placed at position (b) (i), (c) (ii), (d) (iii) placed simultaneously, (e)-(g) Decomposition of RB dye in 15 min time interval, (h)-(j) % decomposition of RB dye in 60 min.

Subsequently, all three beakers were simultaneously placed at positions (i), (ii), and (iii) as shown in Fig. 5 (a). The % degradation efficiencies decreased further at positions (i) and (ii) compared with the single-beaker configuration, suggesting redistribution of acoustic intensity among the three-glass beakers. Nevertheless, position (iii) remained the least active location, confirming that it consistently experiences the lowest acoustic pressure, as can be seen in Fig. 5(b)-(d).

The influence of dye volume was then examined using 20-60 mL RB solution while maintaining the three-beaker configuration as presented in Fig. 5(e)-(j). In all cases, degradation decreased as solution volume increased. Interestingly, degradation became nearly constant above 50 mL, indicating that 50 mL is sufficient to achieve reproducible degradation in a 100 mL glass beaker.

**Fig. 6:**
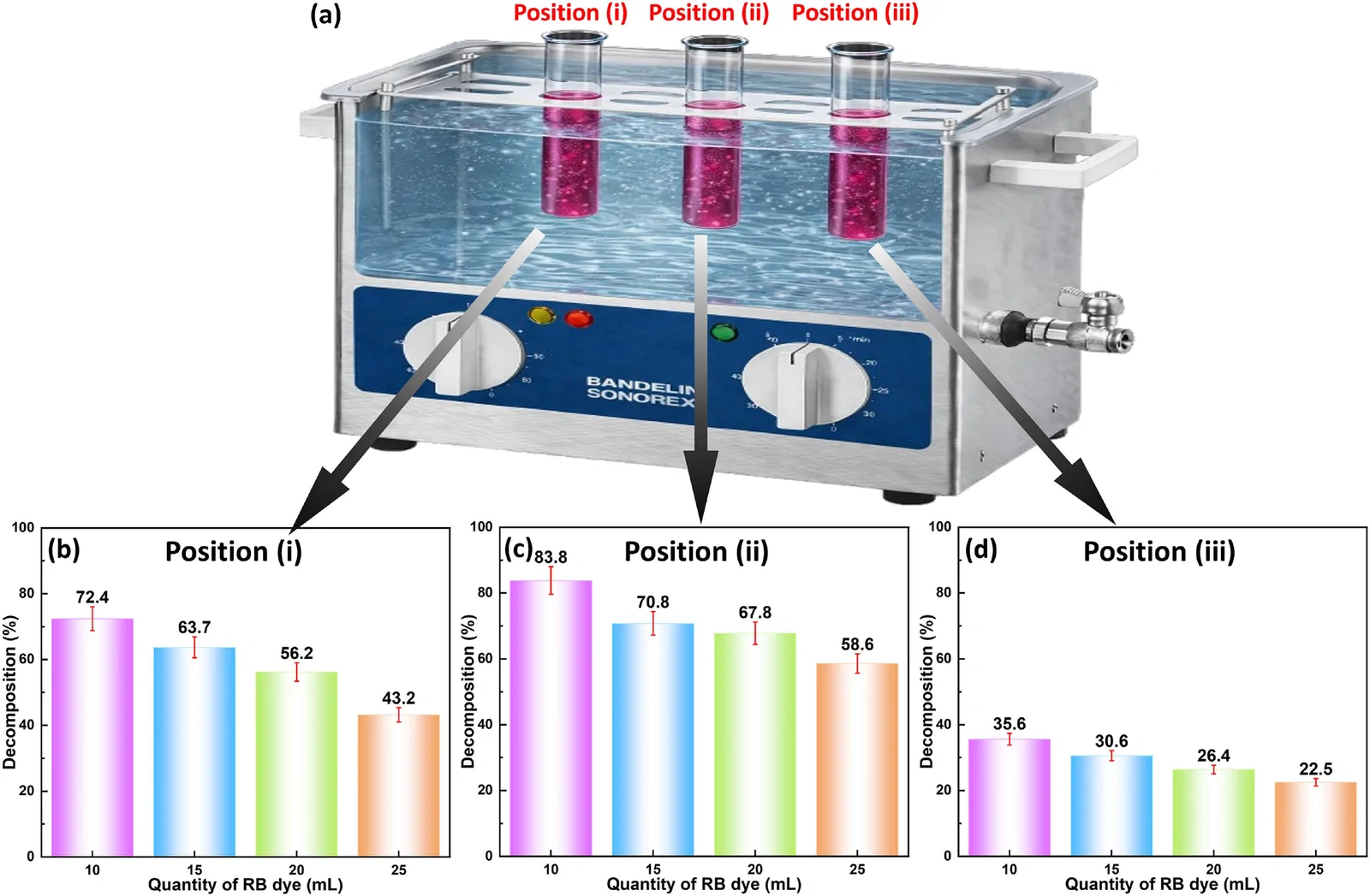
(a) Schematic representation of the glass tube placed directly in the water bath, (b)-(d) degradation of RB dye (10, 15, 20, and 25 mL) at positions (i), (ii), and (iii).

**Fig. 7:**
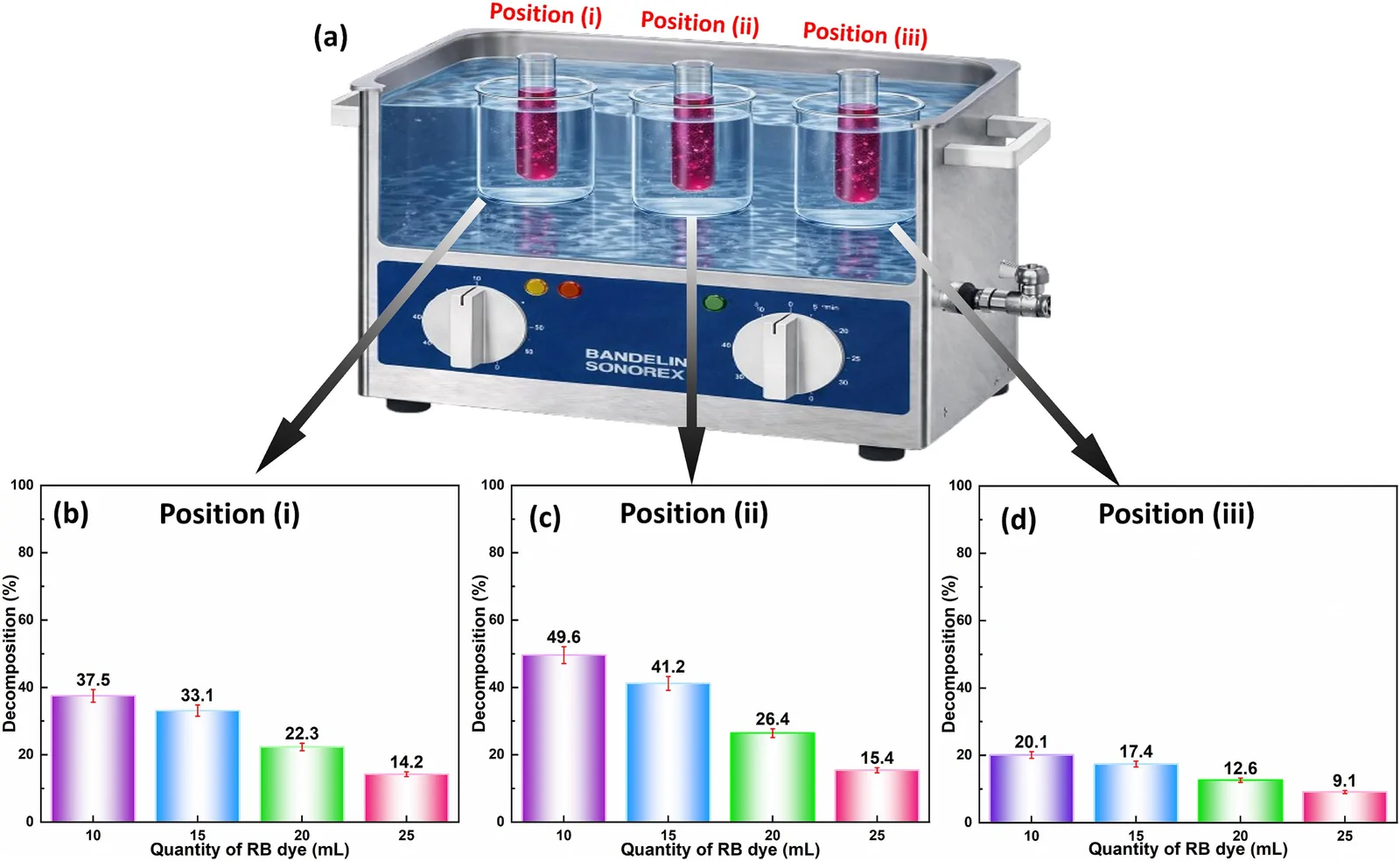
(a) Schematic representation of the glass tube in a water-filled glass beaker placed in an ultrasonicator, (b)-(d) degradation of RB dye (10, 15, 20, and 25 mL) at positions (i), (ii), and (iii).

To investigate the influence of reactor geometry, the experiments were carried out using glass tubes (30 mL) simultaneously placed at positions (i), (ii), and (iii), as shown in Fig. 6(a). The results shown in Fig. 6(b)-(d) revealed that, compared with glass beakers, significantly higher degradation was observed under identical ultrasonic conditions. However, the positional dependence remained unchanged, with position (iii) exhibiting the lowest degradation. Finally, the glass tubes were placed inside water-filled glass beakers as presented in Fig. 7 (a). Under this condition, the obtained results are presented in Fig. 7(b)-(d), and the degradation decreased substantially compared with that observed for the tubes placed directly in the ultrasonic bath. The additional water-glass-water interfaces introduced further reflection, transmission losses, and attenuation of ultrasonic waves before they reached the dye solution. The physical origin of these observations is discussed in 4.2, 4.3. The results obtained in the RB dye degradation investigations are summarized in Table 1, Table 2, Table 3.

**Table 1:** Summary of RB dye degradation (60 min), compiled from Figs. 1–3.

Configuration (glass beaker 50 mL)	Position (i)	Position (ii)	Position (iii)	Observations
Single beaker	22.1 %	23.6 %	6.4 %	Degradation at position (iii) is minimum.
Two beakers (position (i)/(ii))	15.4 %	17.6 %	−	Degradation at positions (i) and (ii) decreased compared to single beaker placement.
Two beakers (position (ii)/(iii))	−	23.5 %	2.9 %	Degradation at position (iii) remains minimum.
Two beakers (position (i)/(iii))	15.5 %	−	2.6 %	Degradation at position (iii) remains minimum.
Three beakers (position (i)/(ii)/(iii))	13.5 %	16.1 %	3.1 %	Degradation at each position decreases compared to single beaker placement, while degradation at position (iii) remains minimum.

**Table 2:** Effect of volume on RB dye degradation in three-beaker configuration.

RB dye volume (mL)	Position (i) (in %)	Position (ii) (in %)	Position (iii) (in %)
20	51.3	83.5	25.5
30	23.2	36.5	12.4
40	15.3	34.1	11.1
50	13.5	16.1	3.1
60	13.0	14.4	2.9

**Table 3:** RB dye degradation at positions (i), (ii), and (iii), compiled from Figs. 4–5.

Reactor	Volume (RB dye)	Position (i) (in %)	Position (ii) (in %)	Position (iii) (in %)	Observations
Glass tube	25 mL RB dye	43.2	58.6	22.5	Higher degradation due to greater acoustic confinement and cavitation.
Glass tube inside a water-filled glass beaker	25 mL RB dye	14.2	15.4	9.1	Additional water–glass interfaces attenuate the acoustic waves, reducing degradation.

### 4.2 Behavior of acoustic waves

In an ultrasonicator, there is intrinsically a non-uniformly distributed acoustic field as a result of superimposed incident and reflected waves, and the wave propagating in the longitudinal direction will follow the equation (1). It is assumed that two transducers in the ultrasonicator produce the same pressure intensity simultaneously, which are superimposed, and that the following phenomenon occurs due to the restricted path of the glass beaker, producing a varying acoustic field within the water bath. The restriction imposed by the glass beaker will unnecessarily lead to wave interference, resulting in standing waves. Therefore, for two counterpropagating waves of initial equal amplitude, superposition will be in accordance with equation (2) [43]. Furthermore, the intensity (*I*) of the acoustic waves is directly proportional to the pressure amplitude (equation (3), resulting in spatial variation in intensity within the water bath.

(1) $$p\left(x,t\right)=p_{o}\sin\left(\omega t-kx\right)$$

(2) $$p\left(x,t\right)={2p}_{o}\sin\left(\omega t\right)cos\left(kx\right)$$

(3) $$I\left(x\right)\propto {cos}^{2}\left(kx\right)$$

Here, $p_{o}$, ω, k, x and t represent the pressure amplitude, angular frequency, wave number, direction of its propagation, and time, respectively.

**Fig. 8:**
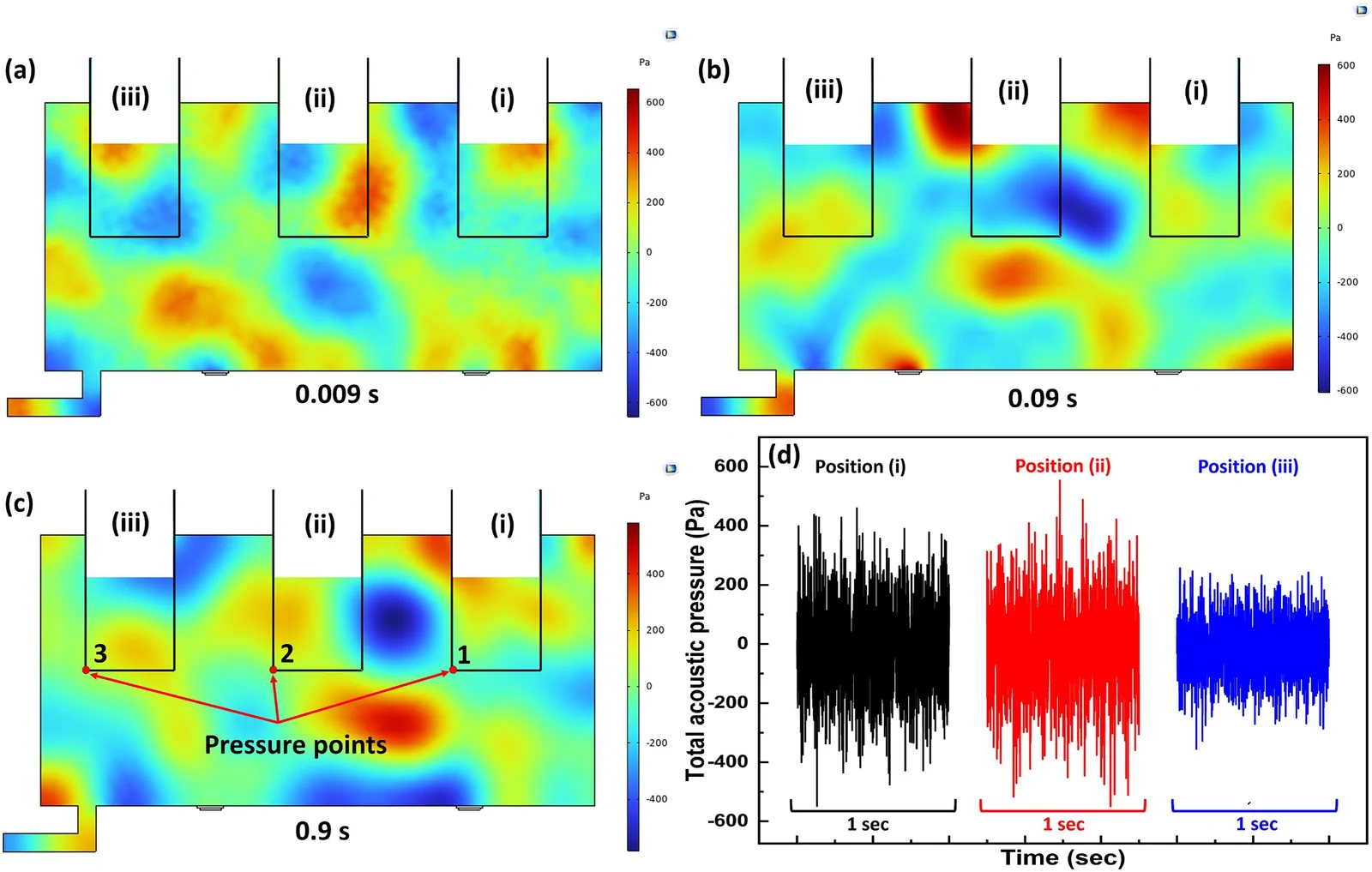
Acoustic pressure field in water bath and 50 mL water filled in 100 mL glass beaker at time (a) 0.009 s, (b) 0.09 s, and (c) 0.9 s, (d) acoustic pressure at points 1, 2 and 3 inside glass beaker positioned at (i), (ii) and (iii) over a time duration of 1 s.

From the individual and combined beaker placements, one thing is clear: the beaker's position within the ultrasonicator's domain is pivotal. The distinct dye degradation at position (iii) and (i)/(ii) is primarily due to such non-uniformly distributed acoustic waves within the ultrasonicator domain. Thus, at certain points, for a very short time, the local pressure ($P_{\mathrm{local}}$) in the dye solution drops below its vapor pressure $\left(P_{\mathrm{vp}}\right)$, hence forming bubbles that eventually expand to burst in areas of higher pressure. Acoustic cavitation occurs only when the magnitude of the negative local pressure exceeds the cavitation threshold ($P_{\mathrm{local}}$ > $P_{\mathrm{cavitation}}$). Therefore, only those regions contribute to cavitation-induced radical generation. Conceptually, the effective cavitation volume can be expressed as $V_{\mathrm{cav}}=\int dV$, over all the regions satisfying the condition, $P_{\mathrm{local}}$ > $P_{\mathrm{cavitation}}$. From the experimental results with the RB dye-filled glass beaker and tube, the least degradation was attained at position (iii). The reason is due to spatial non-uniformity and low-pressure reaching position (iii). To visualize the non-uniform acoustic pressure within the water bath and the liquid in the glass beaker, a computational simulation was conducted using COMSOL *software*, as shown in Fig. 8(a)-(d). The pressure variation was visualized in the water bath, which represented liquid (water here). The computational study was conducted using the exact dimensions of the ultrasonicator used and three glass beakers of the same dimensions. The pressure variations within the water bath and in the water-filled glass beaker at random times 0.009 s, 0.09 s, and 0.9 s are presented in Fig. 8(a)-(c). Further, the pressure variation at points 1, 2, and 3 is shown in Fig. 8(c) at positions (i), (ii), and (iii), and is presented in Fig. 8(d) for a time interval of 1 s. The intensity of pressure at position (iii) was less compared to positions (i) and (ii). The computational and experimental study complements each other, showing a variation of pressure within the dye contained in the glass beaker, where the least pressure is reached at position (iii), which is responsible for the least degradation at position (iii). An additional observation from the sonolysis experiments was that, when the solution volume was below 50 mL, higher degradation was accompanied by greater variation in the obtained results. Beyond a solution volume of 50 mL, the dye degradation remained nearly constant.

This behavior could be due to the modifications in the acoustic field owing to the liquid level. At lower solution volumes, the liquid occupies a smaller portion of the acoustic field, which may alter the local acoustic energy distribution and cavitation activity, resulting in greater degradation. The acoustic field may also become more sensitive to changes in liquid height and reflections at the liquid-air boundary, causing variations in the cavitation volume and affect reproducibility. At 50 mL, the acoustic conditions appear to become relatively stable, such that further increases in solution volume have a limited effect on the effective cavitation region, resulting in nearly constant degradation and better reproducibility. In this regard, previous studies have indicated that sonochemical activity can be decreased with rising liquid level during laboratory-scale investigations, highlighting the dependence of cavitation activity on solution volume [44], [45]. This behaviour suggests that 50 mL represents the minimum solution volume required to obtain relatively stable and reproducible degradation under the present ultrasonic bath configuration. This observation is consistent with previous studies showing that cavitation activity depends on the local acoustic pressure distribution, acoustic wave attenuation, and reactor geometry rather than solely on the applied ultrasonic power [46], [47].

The experiments also showed that, for the same amount of dye, greater degradation occurred when the solution was placed in a glass tube (10 cm height, 2.5 cm diameter) than in a glass beaker (7 cm height, 5 cm diameter). This difference may be associated with the different reactor geometries, including the liquid height, diameter, headspace, and volume-to-surface ratio, which can modify the distribution of acoustic energy within the liquid [48]. A smaller diameter or headspace in the glass tube might restrict the acoustic energy to a smaller volume of liquid, which could produce higher acoustic pressures and cause more cavitation, thereby leading to higher sonolytic degradation. On the other hand, the large diameter and volume of the glass beaker might dissipate the acoustic energy to a larger area, hence producing lower cavitation effects.

### 4.3 Interaction of the acoustic waves with glass material (liquid-solid interface)

When a glass beaker is placed inside the water bath, the wave propagation will experience different phenomena (reflection, absorption, and transmission) [49]. For instance, from a water bath to a glass beaker to the dye present in it. Therefore, there will be two interfaces: liquid-solid (water bath to the outer glass beaker wall) and solid-liquid (the inner glass beaker wall to the dye). At the interface, there will be a mismatch in acoustic impedance [49], where the acoustic impedance (Z) is defined as the product of the medium's density (ρ) and the speed of sound (c) in that medium ,Z=ρc. Initially at the liquid–solid interface, some of the part of the wave will be reflected and transmitted, thus at any normal incidence on the outer glass wall, the reflection (R) and transmission (T) coefficients will be $R=\frac{Z_{2}-Z_{1}}{Z_{2}+Z_{1}}$ and $T=\frac{2Z_{2}}{Z_{2}+Z_{1}}$ (here, $Z_{1}$ and $Z_{2}$ refer to the acoustic impedance of the water bath and glass beaker) [49]. The majority of acoustic waves are reflected at the glass's interface, bouncing back rather than entering the glass medium because$Z_{2}$ > $Z_{1}$. The transmitted waves entering the glass beaker undergo attenuation, primarily due to viscoelastic losses and scattering from structural inhomogeneities [50]. Thus, the acoustic waves traveling through the water bath undergo superposition after reflection from the two transducers. Then, enter the glass beaker, experience attenuation, and travel back into the dye solution, where it encounters a spatial pressure difference. In general, the effects of reflection, transmission, and superposition produce a highly spatially heterogeneous distribution of the pressure waves. Since the magnitude of the acoustic reflection coefficient increases with increasing acoustic impedance mismatch between two media, a hypothetical reactor fabricated from a dense material, such as ceramic, for instance, would possess a significantly higher acoustic impedance than water. A larger Z would increase the reflected component of the incident ultrasonic wave [51] and correspondingly a smaller fraction would be transmitted into the reaction solution. Furthermore, attenuation and scattering within the ceramic wall would reduce the acoustic pressure reaching the liquid, leading to weaker cavitation and lower sonolytic degradation, and to low piezocatalytic activity due to less vibration reaching the dye solution. Conversely, if the reactor is of polymeric material, its Z would generally be closer to that of water [52], thereby reducing the impedance mismatch and increasing the transmitted component of the incident ultrasonic wave. However, polymers are viscoelastic materials and exhibit considerably higher internal damping [53]. Consequently, a significant fraction of the transmitted acoustic energy would be dissipated within the polymer wall before reaching the liquid, thereby reducing the effective pressure amplitude available for cavitation. Therefore, although impedance matching improves wave transmission, the accompanying material attenuation may offset this advantage. These proposed examples demonstrate that the acoustic field inside a sonochemical reactor is also governed by material attenuation.

The above discussions clearly indicate that when the dye-filled glass tube is placed in a water-filled beaker, RB dye degradation is lower than when the glass tube is placed directly in the ultrasonicator bath. This indicates the influence of the reactor material, as the acoustic wave has to travel twice through the glass material before reaching the RB dye contained in the glass tube. The degradation of the individual and combined (for both two- and three-beaker positions) is reduced when all three beakers are kept together. This could be attributed to redistribution of acoustic intensity within the bath when three beakers were placed simultaneously and degradation trend became more uniform. Therefore, the study was continued by simultaneously placing all three beakers, and the piezocatalytic activity of the NBT powder was investigated.

### 4.4 Piezocatalytic investigations using NBT ceramics

**Fig. 9:**
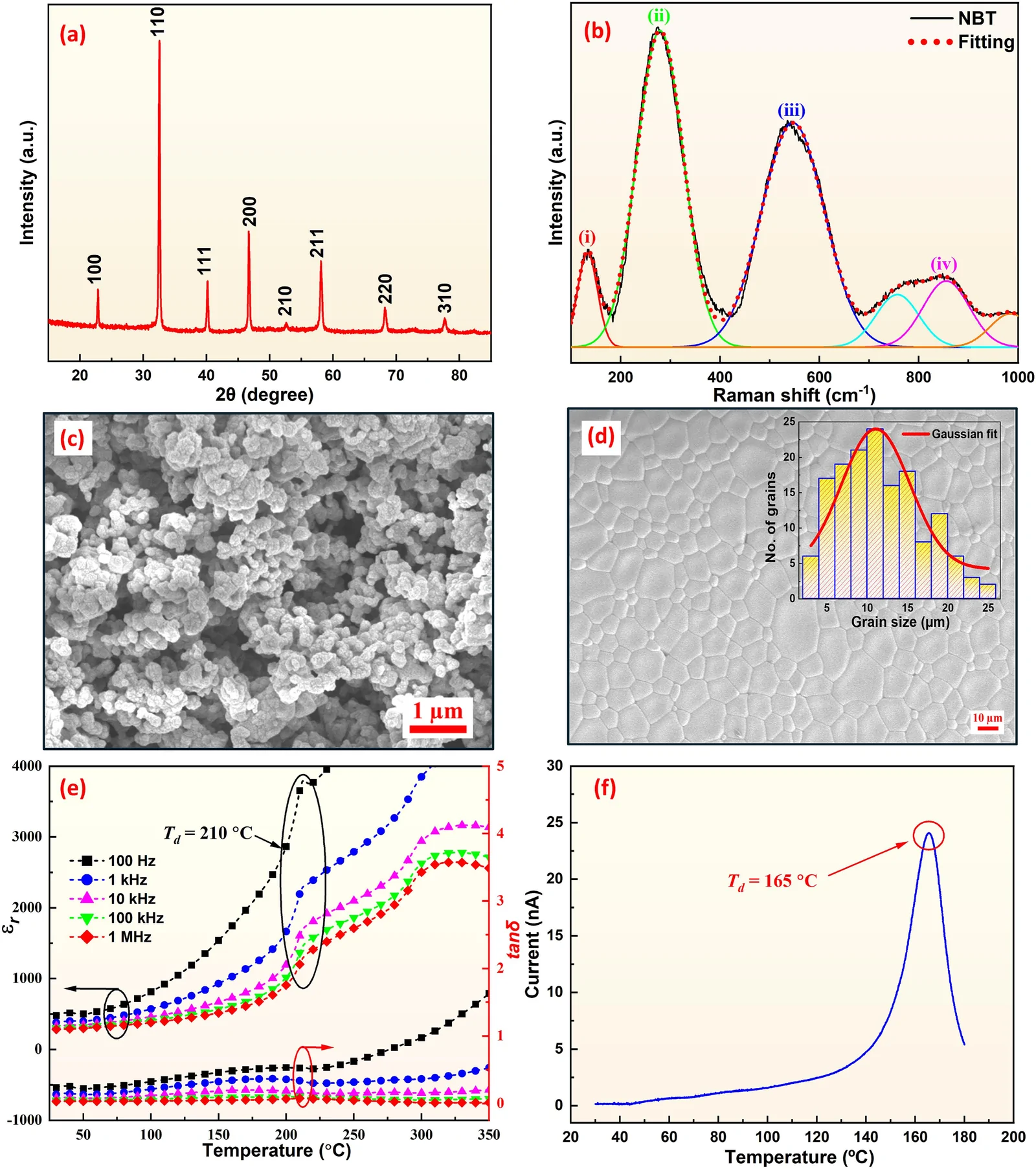
(a) XRD pattern in 2θ degree range of 20-80°, (b) Raman spectroscopy, (c)-(d) SEM image of powder and bulk pellet, (e) dielectric characteristics, and (f) Pyroelectric current of NBT ceramics.

Before conducting the dye degradation experiments using the catalyst, NBT powder was characterized. Fig. 9(a) shows the XRD pattern of the NBT ceramic synthesized in the 2θ range of 20-80°. The pattern is consistent with ICDD file 36-0340, revealing rhombohedral symmetry and the purity of the NBT ceramic powder [54]. The Raman pattern in the 50-1000 cm^1^ wavenumber range (Fig. 9(b)) provided details on vibrational modes in the NBT powder. Following the deconvolution of NBT powder's spectrum, four major peaks in the range of 50-200 cm^−1^, 200-400 cm^−1^, 400-750 cm^−1^, and 750-1000 cm^−1^ were observed relating to Na-O, Ti-O, TiO_6_ octahedral, and due to oxygen vacancies, respectively [55]. The modes in the spectrum correspond to the NBT's rhombohedral structure, and, accordingly, the Raman spectrum agrees with the XRD pattern, validating the purity of the NBT ceramic powder.

Fig. 9(c) and (d) show the SEM images of the powder and the pellet. The powder image is presented at a 1 µm scale, with particles ranging from 150 nm and mostly irregular or elongated ovals. Moreover, the average grain size of the NBT ceramic pellet is ∼ 10-15 µm as shown in Fig. 9(d). NBT ceramic, being dielectric in nature, possesses a ferroelectric nature, as enforced by the temperature-dependent dielectric investigation. The dielectric permittivity (ɛ*_r_*) and dielectric loss (tan*δ*) of NBT were measured at 100 Hz, 1 kHz, 10 kHz, 100 kHz, and 1 MHz in the temperature range of 30 °C to 350 °C, as presented in Fig. 9(e). The value of depolarization temperature (*T_d_*) is 165 °C from pyroelectric measurements, as shown in Fig. 9(f). Further, the value of *d_33_* for the poled NBT pellet was found to be ∼ 59 pCN^−1^, which demonstrates the effective piezoelectric response of the synthesized NBT ceramic [56].

**Fig. 10:**
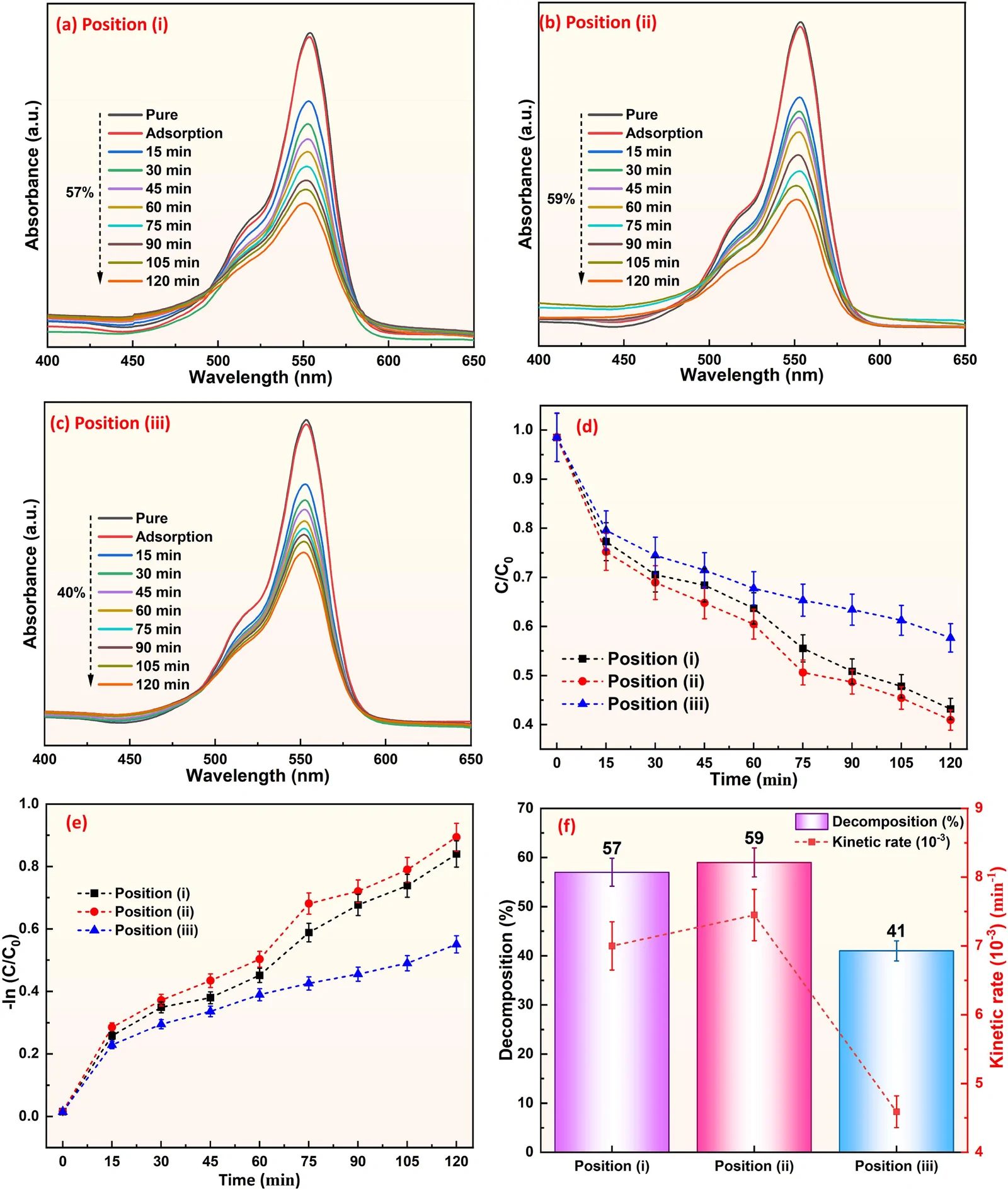
UV–vis absorbance spectra of RB dye degradation induced by ultrasonication using NBT powder (a) position (i), (b) position (ii), and (c) position (iii), (d) plot of $C/C_{0}$*vs* time, (e) plot of $\ln\left(C/C_{0}\right)$ with time, and (f) comparison of decomposition efficiencies and kinetic rate of RB dye for NBT after 120 min.

Fig. 10 (a)-(c) shows the absorbance spectrum of the RB dye using NBT catalyst at positions (i), (ii), and (iii) simultaneously at the same time. Fig. 10(d-e) represents $C/C_{0}$ and $\ln\left(C/C_{0}\right)$ *vs* time plots showing the % degradation at every 15 mins and the kinetic rate of the degradation, respectively. Fig. 10(f) shows the % degradation of RB dye in 120 mins, which are 57 %, 59 %, and 41 % at positions (i), (ii), and (iii), respectively. Fig. 10(f) also presents the degradation rate, calculated to be 7 × 10^-3^, 7.45 × 10^-3^, and 4.95 × 10^-3^ min^−1^. From the piezocatalysis investigation, it can be seen that the least degradation was observed at position (iii). However, if we see the investigations of sonolytic and piezocatalytic degradation, a similar observation of the least RB dye degradation at position (iii) is observed.

Following the piezocatalytic investigations using NBT, another important question arises: can the degradation obtained without a catalyst (sonolysis) be directly subtracted from the degradation obtained during NBT-assisted piezocatalysis at positions (i), (ii), and (iii) to determine the true piezocatalytic activity of the NBT ceramics? Under ultrasonic conditions, acoustic cavitation and the piezoelectric effect occur simultaneously during the piezocatalytic process and are inherently coupled. To examine the influence of acoustic cavitation, an experiment was performed by introducing fine sand particles into the sonolysis system. The results obtained using sand particles in the RB dye degradation are presented in Fig. S1 (Supplementary Information). Compared with sonolysis degradation, degradation decreased in the presence of sand, which can be attributed to ultrasonic attenuation, acoustic (bubble) shielding, reduced collapse of cavitation bubbles near the pollutant surface, and physical shielding of the generated reactive species. These results demonstrate two major findings: first, the cavitation-induced contribution is reduced in this particular case by using sand particles; second, there is still some contribution from acoustic cavitation leading to RB dye degradation. Similarly, in the piezocatalytic system, the presence of the ceramic catalyst is expected to affect the cavitation field and simultaneously generate reactive species via the piezoelectric effect. Therefore, the observed degradation during piezocatalysis arises from the intrinsic piezocatalytic activity, together with a reduced but unavoidable contribution from acoustic cavitation. Accordingly, the present approach minimizes the sonolytic contribution through experimental design, providing a more representative evaluation of the intrinsic piezocatalytic activity of the ceramic material.

## 5 Suggested experimental steps for minimizing sonolysis

Increasing ultrasonic frequency tends to reduce the intensity of transient cavitation because bubbles have less time to grow and collapse violently, favoring more stable cavitation and reducing radical generation, although the effect can vary depending on the reaction system [57]. Moreover, with increased acoustic power, cavitation and sonochemistry improve up to an optimal value, after which high cavitation forms more bubbles and acts as a barrier to the propagation of acoustic power in the reactor [57]. Furthermore, higher acoustic power leads to larger bubbles, while frequency is inversely proportional to bubble size; hence, both frequency and power affect cavitation intensity, bubble behavior, and sonochemistry. In particular, for dye degradation applications, Franziska Bößl *et al.* [42] examined the effects of frequency (20 kHz to 1 MHz) and power (∼3 to 90 W L^−1^), finding that at low frequencies (<100 kHz), the mechanical effects prevailed. With increasing ultrasonic power, the dye degradation process improved. It should be noted that the results obtained during the current study should be considered within the framework of the investigated ultrasonic parameters (5.5 L, 35 kHz, 160 W) in a single bath-type ultrasonicator. In the case of the current system, the optimal sample position is position (ii), and the critical solution volume was found to be 50 mL for 100 mL glass beaker. Nevertheless, the primary contribution of this work is the establishment of a reactor-specific calibration framework rather than universal operating parameters. Future studies should utilize this methodology across different ultrasonic systems before conducting piezocatalytic investigations. Here are some steps researchers can follow to avoid background effects in piezocatalytic dye degradation applications using laboratory-scale frequency/power sonicators.

A) Identify the optimum position for sample placement. This can be achieved by maintaining a spacing equal to the combined diameter of the reactors. In the present study, the ultrasonicator bath is divided into three equivalent sections.

B) Once the reactor position is fixed, optimize the solution volume by determining a threshold volume above which the degradation does not increase further.

C) Maintain identical reactor geometry, reactor position, and solution volume throughout all subsequent experiments.

D) The same ultrasonicator parameters (frequency, power and water bath level) should be maintained throughout the experiments to ensure consistent experimental conditions.

## 6 Conclusions

The present study highlights the contribution of self-degradation, or sonolytic degradation, to the piezocatalysis process by varying the positions of the tested samples within the ultrasonicator's acoustic field. The study further highlights that spatial positioning within the ultrasonicator significantly influences degradation efficiency due to dynamic interactions with the acoustic field. To conduct acoustic-field-affected degradation studies, the tested samples were positioned at different locations: individually, in pair, and in groups of three. From the study, the least degradation was observed at position (iii); furthermore, the same trend of least degradation was observed at combinational positioning. This is attributed to the least vibrations reaching position (iii). Such an effect of the least pressure observed at position (iii) is also validated computationally. The results show a strong dependence on positioning and strongly suggest that the tested sample's positioning should be optimized. Through degradation experiments, it can also be concluded that there is a minimum pollutant volume that needs to be optimized for particular type of reactor geometry; below this volume, the dye's degradation may vary unevenly and considerably. Overall, the present study demonstrates the importance of the positioning and volume of the tested samples for detecting piezocatalyst’s apparent activity. This study further highlights that, prior to investigating piezocatalytic activity, the positioning of the tested sample and the critical pollutant volume should be established as mandatory steps. The procedure followed will be instrumental in ensuring repeatability in piezocatalytic investigations.

## CRediT authorship contribution statement

**Mohit Singhal:** Writing – original draft, Validation, Methodology, Investigation, Data curation, Conceptualization. **Akshay Gaur:** Writing – review & editing, Visualization, Conceptualization. **Brajesh Kumar Nagar:** Data curation. **Dhiraj Kumar Singh:** Data curation. **Satyanarayan Patel:** Writing – review & editing, Visualization, Supervision, Resources, Methodology, Investigation, Funding acquisition, Conceptualization.

## Funding

S.P. thanks the Science and Engineering Research Board (SERB) for financial support in the frame of the Core Research Grant no. CRG/2023/000374. This work is also supported by the PAIR Network of SAKSHAM, funded by ANRF (Anusandhan National Research Foundation), India, through grant ANRF/PAIR/2025/000018/PAIR, administered and mentored by IIT Indore. A.G. would like to thank Anusandhan National Research Foundation (ANRF) and its funding PDF/2025/000357 for conducting the study.

## Declaration of competing interest

The authors declare that they have no known competing financial interests or personal relationships that could have appeared to influence the work reported in this paper.
