# Supplementary material for: Influence of sample positioning and solution volume on distinguishing sonolysis from piezocatalysis in ultrasonic dye degradation
Source: Ultrason Sonochem. 2026 Sep 10;133:108049. doi: 10.1016/j.ultsonch.2026.108049 (PMC13599587; doi:10.1016/j.ultsonch.2026.108049)
Supplement: Supplementary file 1 [file mmc1.docx]

**Supplementary Information (SI)**

**Influence of sample positioning and solution volume on distinguishing sonolysis from piezocatalysis in ultrasonic dye degradation**

Mohit Singhal, Akshay Gaur, Brajesh Kumar Nagar, Dhiraj Kumar Singh, and Satyanarayan Patel*

Department of Mechanical Engineering, Indian Institute of Technology Indore, 453552, India

*Corresponding author: [spatel@iiti.ac.in](mailto:spatel@iiti.ac.in)

**Figure S1:** Absorbance spectrum of RB dye at position (a) i,(b) ii, (c) iii, using sand particle and (d)% degradation of RB dye with and without sand at positions (i), (ii) and (iii).
